# Threats & Trade-offs: A Start-up Simulation Game for Cybersecurity and Innovation Decision-Making

**DOI:** 10.1007/s10796-025-10604-3

**Published:** 2025-04-22

**Authors:** Kseniya Stsiampkouskaya, Oishee Kundu, Joanna Syrda, Adam Joinson

**Affiliations:** 1 School of Management, University of Bath, Claverton Down, Bath, BA2 7 AY UK; 2 Present Address: School of Social Sciences, Cardiff University, Cardiff, CF24 4HQ United Kingdom

**Keywords:** Cybersecurity, Game-based learning, Serious games, Digital secruity, Business management

## Abstract

Cybersecurity is now critically important in an increasingly digitized and connected world. In addition to required digital security, individuals and organisations pursue multiple other objectives under binding resource constraints. Understanding how they make decisions in the face of these trade-offs is important for both research and teaching purposes. Games can create effective and exciting learning environments and also provide an immersive and experiment-based research setting to understand decision-making. We present a novel tabletop board game which sets cybersecurity in a broader organisational context and emulates real life business decisions. It can be used as a powerful research tool to understand decision-making about cybersecurity in a resource-constrained and uncertain environment. It is also a useful interdisciplinary educational tool, integrating concepts from cybersecurity, business development, and innovation management in gameplay.

## Introduction

The ubiquity of digital products and services in public and private life has increased the importance of cybersecurity for individuals, businesses, and society. A high demand for a workforce skilled in cybersecurity has resulted in the creation of novel pedagogical tools (Daniel et al., 2023), as well as a growing body of research on factors affecting cybersecurity decision-making (Ceric & Holland, 2019; Safi & Browne, 2023; Shreeve et al., 2023). Game-based learning has been employed frequently to build awareness and provide training about cybersecurity (Allal-Chérif et al., 2016; Connolly et al., 2012; Denning et al., 2013). However, existing cybersecurity games tend to focus on information systems and cybersecurity in a silo, without considering trade-offs that may arise due to other priorities like business development or operational management, or assuming a fixed and dedicated cybersecurity budget. Not all cybersecurity investment models take the opportunity cost of product development or marketing into account, which is a critical consideration for innovative start-ups and SMEs (Heidt et al., 2019).

In this paper, we present an original tabletop boardgame called ‘Threats and Trade-offs'(T&T) which places cybersecurity decision-making in the context of a digital technology-intensive start-up, combining digital security considerations with business management and innovation. In the game, groups of players imagine themselves to be key decision-makers in an emerging technology company. They face various cyber threats over the course of the game and make decisions between investing in cybersecurity, product innovation, and growing their customer base. The game design is grounded in theoretical models of business strategy and cybersecurity and is an interactive and engaging tool that (1) increases understanding of cybersecurity, including different cyber-attacks, their consequences and countermeasures; (2) improves knowledge of business development, including product innovation, customer acquisition, and customer and employee satisfaction management; and (3) reveals decision-making on cybersecurity adoption under constraints and competing demands.

In the next section, we provide a review of literature on cybersecurity investment, particularly focussing on the use of game-based learning for cybersecurity and behavioural research on cybersecurity investment. Section 3 describes the game and its elements. Section 4 presents evidence from a playtest that was organised with 32 postgraduate students enrolled in a cybersecurity course at a British university. The evidence highlights the game’s playability, player experiences, and discussions that emerge from playing the game. The final section summarises our work and discusses future directions of research and teaching involving this board game. The paper is also an invitation to other members of the information systems and digital security community to critically engage with the boardgame as a research and educational tool for their own purposes and the resources can be accessed at https://www.discribehub.org/boardgame-threats-tradeoffs.

## Background

The increasing adoption of ICT technologies by businesses, especially since Covid- 19 (Wendt et al., 2022), makes the ubiquity of the digital world undeniable. As society becomes more digitally reliant, the importance of cybersecurity increases. Cybersecurity investment by businesses is often motivated by the high cost of cyber breaches (Shaikh & Siponen, 2023) as well as the interdependencies between firms sharing common networks or along the supply chain (Ezhei & Tork Ladani, 2020; Fedele & Roner, 2022). Given all the possible yet uncertain costs it is difficult to identify an optimal level of investment. Investment decisions are even more challenging for resource-constrained SMEs and start-ups, what Heidt et al. (2019) poignantly characterize as “the Security Divide” between organisations based on their size.

Cybersecurity measures can be technology-based and human-based (i.e., measures like employee training), and successful cyberattacks tend to exploit the human element more often (McAlaney & Benson, 2020; Verizon, 2023). In response to this, innovative educational and training programmes have been developed to impart cybersecurity skills and knowledge (Daniel et al., 2023; Foreman et al., 2015; Stites et al., 2013; Yardley et al., 2014), with a growing emphasis on game-based learning for cybersecurity (Allal-Chérif et al., 2016; Cornel et al., 2017; Denning et al., 2013; Karagiannis & Magkos, 2021). Gameplay has also been used to conduct behavioural research on cybersecurity decision-making (Frey et al., 2019; Safi & Browne, 2023; Shreeve et al., 2022). We briefly review the two strands of literature, providing an overview and highlighting the knowledge gap and problem that we seek to address with our new game.

### Game-Based Learning for Cybersecurity

At the very outset, it is important to distinguish between ‘gamification’ and ‘game-based learning’ (see Karagiannis & Magkos, 2021). ‘Gamification’ attempts to turn the learning process into a game by introducing elements from games like turns, rewards, strategy, role-play, etc., while ‘game-based learning’ (GBL) means bringing a game into the learning environment. We focus here on the latter (GBL) and not the former.

Educational boardgames exist in many different subject areas, including cybersecurity, and it has been found that games produce greater engagement and better learning outcomes (Allal-Chérif et al., 2016; Connolly et al., 2012; Denning et al., 2013; Younis & Alghamdi, 2021). For example, Allal-Cherif, et al. (2016) provide a critical review of the use of games to train new employees in the banking sector and find that games achieved higher engagement than other forms of knowledge delivery (i.e., lectures). Their research highlights two unique contributions that games can make in the learning environment: (1) ‘externalisation’ of internal knowledge, as players share their own experiences when they encounter a situation or decision-point in the game, and (2) ‘historization’ of past events in fictional form through game context and storytelling.

Other reviews on the use of games for cybersecurity teaching and training find that games can attract interest from students who might otherwise be intimidated by the knowledge demands associated with the digital world, like programming or mathematics (Anvik et al., 2019; Younis & Alghamdi, 2021). Games can also play a social role in the educational environment, as an icebreaker for new students and as a facilitating tool for socialising outside the classroom like playing the game in the student lounge (Denning et al., 2013). Connolly, et al. (2012) note that “modern theories of effective learning suggest that learning is most effective when it is active, experiential, situated, problem-based and provides immediate feedback”, and games often meet all these requirements.

Weishaupl et al., (2018) refer to cybersecurity incidents as ‘learning triggers’, and most games incorporate security incidents or cyber-attacks to impart knowledge of countermeasures against cyber-attacks. Most pedagogical cybersecurity board games focus on educating players on different cyber-attacks and defences. Games, such as *iMonsters* (Tseng et al., 2022), *{[d0* × *3 d!]}* (Gondree & Peterson, 2013), *Protection and Deception* (Zahir et al., 2015), and *Riskio* (Hart et al., 2020) are designed to increase players’ understanding and knowledge of various cyber risks and ways to avoid them. Due to their introductory nature, these games are aimed at a relatively inexperienced audience with little technical knowledge. Simultaneously, games that are aimed at audiences with more advanced cybersecurity skills, such as *Backdoors & Breaches* (Young & Farshadkhah, 2021), also exist but are less common. In addition to physical tabletop exercises, educational scenario-based cybersecurity video games, such as CyberCIEGE (Thompson & Irvine, 2011), are used for training cybersecurity students and professionals.

Serious games such as community-based tabletop exercises and scenarios have been deployed to build cybersecurity preparedness at many levels. For example, the Federal Emergency Management Agency (FEMA) collaborated with cybersecurity experts to develop Cyber Ready (The Federal Emergency Management Agency, 2025), a cooperative community-based strategy board game designed to dynamically educate players and increase their knowledge and understanding of the National Institute of Standards and Technology’s cybersecurity framework (The National Institute of Standards and Technology, n.d.). Similarly, in June 2024, Cybersecurity and Infrastructure Security Agency (CISA) led an international exercise focused on AI-enabled and AI-related cybersecurity incidents by bringing together companies and several international cyber agencies (Vasquez, 2024). Tabletop exercises highlight the importance of coordination and collaboration for addressing cybersecurity incidents, implying that information security is both a technical and a socio-organisational challenge, requiring both technical and managerial skills (White et al., 2004; Conklin et al., 2006). The challenges of coordination between different organisations in case of an incident (“the fog of war”) is especially interesting to simulate and observe with tabletop exercises (Ottis, 2014). While tabletop exercises are considered effective in nurturing soft skills and enhancing practical, hands-on experience for cybersecurity students (Angafor et al., 2020; Dawson & Thomson, 2018), they are also extremely useful for those already in the real-world. For instance, Bahuguna et al. (2019) highlight that tabletop exercises lead to self-realization of cybersecurity postures, creating lasting impressions and producing actionable insights for participants.

Our paper builds upon this foundation by focusing on the challenges of prioritization and coordination within an organization, particularly in a start-up scenario. We explore how tabletop exercises can expose and address these challenges, emphasizing the need for effective communication and the translation of technical concepts between decision-makers. By simulating real-world business operations contexts, our work aims to enhance the practical application of cybersecurity measures, ensuring that technical knowledge is seamlessly integrated with business management skills to improve overall organizational resilience.

However, there is still a gap regarding coordination and prioritization of cybersecurity *within* an organisation. Most start-ups and SMEs do not have dedicated IT departments and security specialists (Heidt et al., 2019), and cybersecurity training which focuses exclusively on security and ignores other goals in business and management may be insufficient for employees in such organisations. In discussing the use of technologies by SMEs, Ameen, et al. (2022) invite further interdisciplinary work, combining information systems, entrepreneurship, and marketing, and we respond to this call by developing a game that combines learning about cybersecurity with business development. Moreover, there is growing recognition of the multiple roles that individuals play in an organisation, including information security roles, that may be very different from their primary roles but are increasingly becoming “general” and not “specialist” skills (Burns et al., 2021; Posey et al., 2013). Thus, there is a need for cybersecurity and business development games which address multiple learning needs and the complexity of roles and activities in modern digitized organisations.

### Games for Cybersecurity Behavioural Research

There is also a strong body of research on human behaviour and how that can affect preparedness and resilience to cyberattacks in organisations (Beautement et al., 2008; Ceric & Holland, 2019; Crossler et al., 2013; Kweon et al., 2021). A few researchers have applied games and simulations as research tools to unveil cognitive biases and decision-making processes, for example Safi and Browne (2023), Jalali et al. (2019), and Shreeve et al. (2020). Of these, only Shreeve et al. (2020) use a physical boardgame set-up called ‘*Decisions and Disruptions*’.

Games can be used to elicit information from participants (Neag, 2019) and observe collaboration or individual decision-making in teams (Frey et al., 2019; Zeijlemaker et al., 2022). Jalali et al. (2019) use a simulation to observe how decision-makers respond to time delays between cybersecurity investment and its effectiveness, i.e., whether players have a proactive approach to cybersecurity given this delay. Their work suggests that cognitive biases can lead to poor decision making in a complex environment. Safi and Browne (2023) also use a simulation to observe security behaviours like risk monitoring by individuals. Both these experimental settings use the individual as the unit of analysis.

To the best of our knowledge, *Decisions and Disruptions* and *Cyber Ready* appear to be the only games that have been used to understand cybersecurity investment decisions at a group-level. *Cyber Ready* is a strategy game where participants represent specific sectors of community (The Federal Emergency Management Agency, 2025). Players coordinate and cooperate by sharing information and negotiating decisions on how to invest limited resources in different cybersecurity options and defences. *Decisions and Disruptions* requires teams to make decisions about cybersecurity investments in a utility company over successive rounds and observes the choices made by different groups based on their professional backgrounds and experiences (Frey et al., 2019; Shreeve et al., 2022). Investing in specific cyber-defences prevents cyber-attacks which take place at the end of a round, and players are required to decide how to allocate a limited budget across different security measures. The game has several linear attack scenarios that run simultaneously and are not repeatable once they have been prevented. However, in real-life, cyberattacks can repeat and security measures require periodic updates and renewal. Our game involves repeatable cyberattacks, which is explained further in the next section. Furthermore, both *Decisions and Disruptions* and *Cyber Ready* focus on cybersecurity-related decision-making and do not incorporate specific business management opportunity costs to the same extent as *Threats and Trade-offs*.

Additionally, it may be unrealistic to set a fixed “security” budget for SMEs where funds are sporadically dedicated to digital security and are rather allocated between several competing needs and strategic objectives, as confirmed by findings from Heidt, et al. (2019) about the reality of security investment in small firms. Our game responds to this specific set of circumstances by requiring players to make decisions about a set of steering variables (product innovation, marketing, customer and employee satisfaction, security investment, debt management, etc.) simultaneously and providing teams with a dynamic budget which depends on revenue growth and financial losses from cyberattack. From a research perspective, this allows us to observe and analyse cybersecurity investment decisions at different stages of business development and growth. Thus, we add to the repertoire of games that can be used to conduct research on cybersecurity investment behaviour in an organisational context.

## Threats and Trade-offs: a Game of Digital Business Survival

**Fig. 1:**
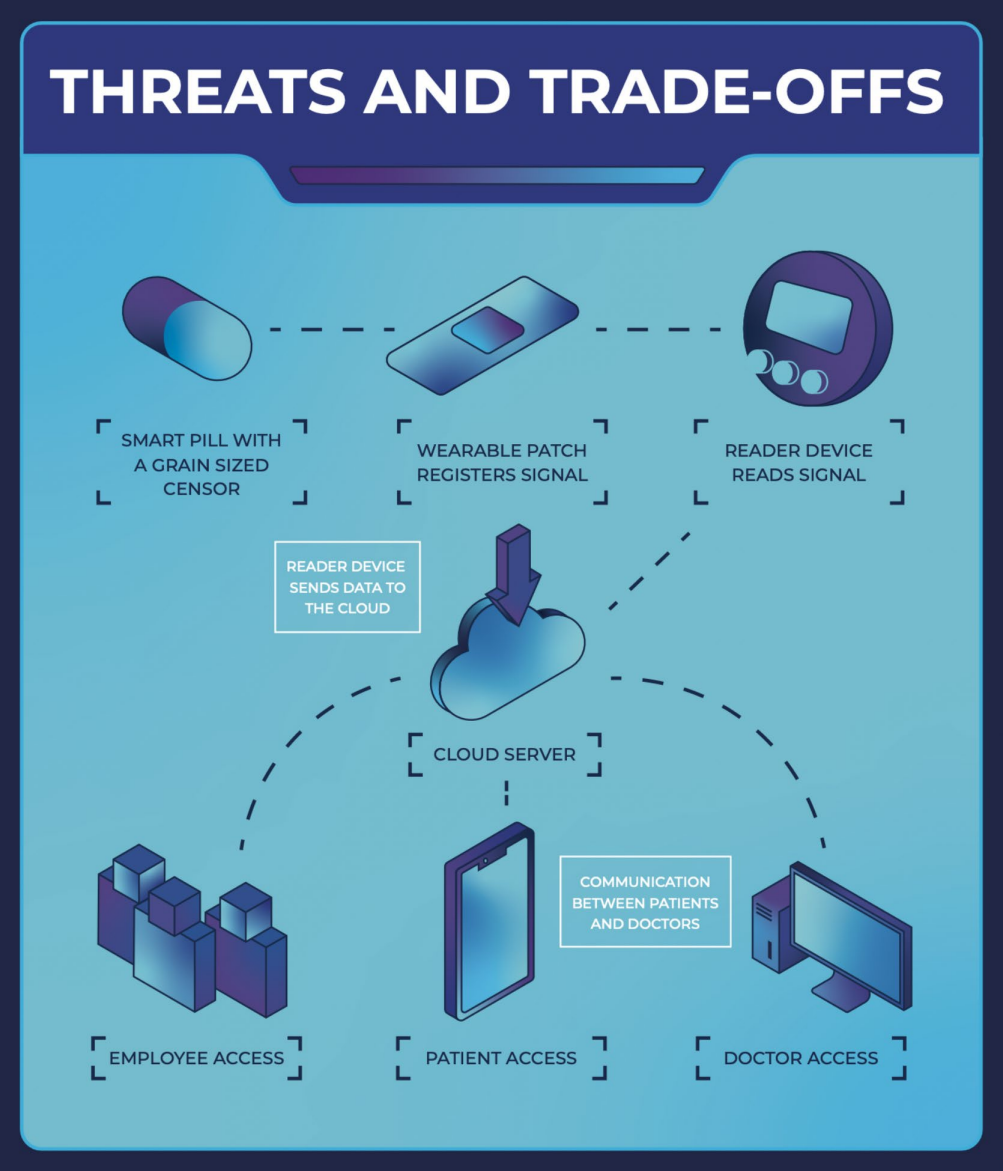
The premise of the game: the dataflow diagram of a connected digital healthcare system

The game conceptualises a connected digital healthcare system, which is inspired by the Abilify MyCite digital ingestion tracking system (U.S. Food & Drug Administration, 2017). The premise (in the form of the business model for the start-up and elements within the system) is as follows (Fig. 1):

1. A patient takes a smart pill whilst wearing an electronic patch on their abdomen.
2. The pill is activated by their stomach fluids and transmits a one-off signal to the electronic patch.
3. The patient uses a reader device (the key product of the game’s start-up) to transfer data from the electronic patch to a cloud database.
4. The data in the cloud database is now accessible to medical professionals through an online portal.

The system is represented by three physical elements (i.e., the pill, the patch, and the reader device). All customer and employee data are stored and processed on a cloud server. The system can be accessed by office employees, doctors, and patients via different interfaces, resulting in diverse initial access opportunities for attackers.

The context of a connected digital healthcare system was chosen to reflect the importance of cybersecurity through the perspective of patient safety and confidentiality (Martin et al., 2017). Additionally, choosing a smart healthcare technology allows us to situate the game in a context where players face multiple challenges, such as developing and marketing a disruptive product in a regulated industry, the complexities and vulnerabilities of advanced technologies, and the lack of customer awareness and willingness to adopt new technologies.

**Fig. 2:**
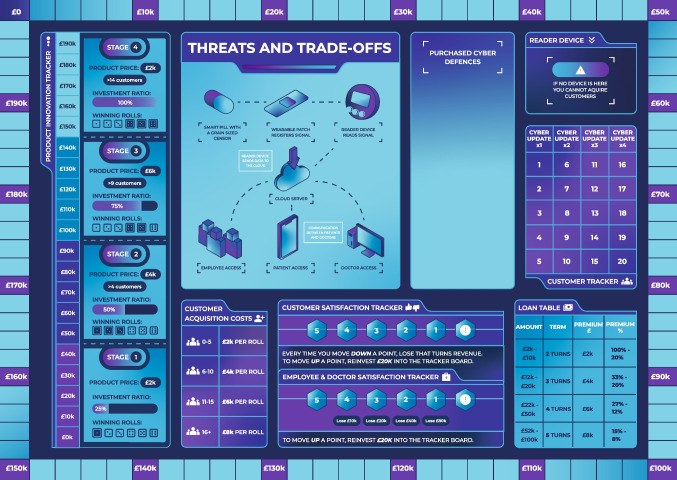
The game board

The game can be played individually (single player) or by a group of up to 8 people playing as a team. The game is played over multiple rounds on a game board (Fig. 2) with three different decks of cards, five counters, and a loan pad. To start a round, the team draws an investor card. They can then make decisions to (1) take a loan, (2) purchase devices and cyber defences, (3) invest in product innovation, (4) invest in customer satisfaction, (5) invest in employee satisfaction, (6) invest in marketing and customer acquisition, and (7) update their cyber defences. A round is not timed, and players can take as many or as few decisions in a round as they wish across all seven categories. To end a round, the team draws a cyber-attack card. If a team does not have the appropriate security measures, they lose customer revenue from the round and face financial and reputational harm (indicated by a fall in the “customer satisfaction tracker” and/or “employee and doctor satisfaction tracker”). Otherwise, they receive customer revenue and can start the next round by drawing another investor card. To win the game, players need to meet the following three criteria for their start-up:

- Achieve a budget of 200,000 in game money.
- Acquire at least 15 customers.
- Have no outstanding loans.

### Game Development

To develop and refine the game, we followed the board game design methods outlined in Fullerton et al. (2004). The process lasted approximately nine months and included the following stages: conceptualisation, prototyping, playtesting, completion, and evaluation. During the initial conceptualisation stage, we completed a review of existing cybersecurity board games and their use cases and benefits, mapping out themes and setting objectives for further work. Simultaneously, we were conducting literature reviews associated with the main themes of the game (i.e., cybersecurity, product innovation, customer acquisition, and business and start-up management). In the prototyping stage, the initial version of the game was created, first as a Power Point presentation of the basic game mechanics and second as a physical paper-based playable copy, followed by approximately six months of playtesting. As a result, the game was developed over the course of four major reiterations, and the final version was refined during eight additional playtesting sessions, each leading to a set of “quality of life” and user experience modifications. The original playtests were conducted within the core research group, supplemented by the consultations with the wider research community (e.g., Digital Security by Design researchers, departmental colleagues, and academic experts in the fields of business and startup management, innovation, and cybersecurity). The additional playthroughs of the final version were conducted with departmental PhD researchers in the fields of cybersecurity and business management. The completion stage included creating a final prototype to hand over to the design team. An additional playtesting session was conducted with the design team to further refine the usability of the game and ensure its accessibility to a non-professional audience. Several reiterations of game design materials were assessed by the core research group in consultation with departmental colleagues. Finally, the game was evaluated during a study described in the Sect. 4 Evaluation and testing.

### Themes

The game encompasses three major themes: cybersecurity, product innovation, and customer acquisition and treats them as the pillars of technology start-up development. Cybersecurity represents the area of threats and uncertainty as the game incorporates a variety of attack scenarios and defence options. Product innovation and customer acquisition represent the business development side of the game as both areas require continuous investment to maintain business growth over time. Furthermore, players must act under the constraints of budget limitations, incomplete information, and high cognitive load which simulate the struggles and challenges of a real-world technology start-up. As a result, players are forced to make complex decisions about cybersecurity and business development which always result in immediate, short-term trade-offs.

#### Cybersecurity

The game incorporates cybersecurity in the form of cyber-attacks and the associated cyber-defences that players can purchase throughout the game to defend their digital infrastructure. The cyber-attack stage happens once per turn, during which players take a card from the attack deck. Each card is associated with a specific attack scenario. Each scenario has countermeasures that prevent it (i.e., cyber-defences) and outcomes that players are subjected to if the attack is not deflected (i.e., cyber-harms) (see Table A1 in Appendix).

**Table 1:** Player characteristics

Variable	Obs	Mean	Std. Dev	Min	Max
Cybersecurity knowledge (0 = very low, 10 = very high)	32	5.438	1.39	2	9
Entrepreneurship knowledge (0 = very low, 10 = very high)	32	5.031	1.636	2	9
Experience playing games or participating in simulations (0 = very little, 10 = a lot)	31	5.645	2.259	1	10

The cybersecurity incidents were selected based on the types of hackers identified by Chng et al. (2022), the STRIDE Threat Model developed by Microsoft (2009), the most common and damaging cyber-attacks according to the Cyber Security Breaches Survey 2022 (Gov.uk, 2022), and MITRE ATT&CK® (n.d.), a global open-access knowledge base that details the tactics and techniques behind a wide range of cyber-attacks and the associated defences used in the used in CIS Community Defense Model 2.0 (Center for Internet Security, 2021). The outcomes of the cyber incidents were identified based on Agrafiotis et al. (2018) taxonomy of cyber-harms and the cyber-attack impacts from the Cyber Security Breaches Survey 2022 (Gov.uk, 2022). Accordingly, MITRE ATT&CK® (n.d.) was also used as a basis for selecting appropriate cyber defences and investment options. The PASTA process (UcedaVelez & Morana, 2015) was used to design relevant scenarios, and a dataflow diagram was created to ensure their viability in the context of a digital healthcare start-up. The scenarios were reviewed by project-external experts conducting research in the fields of traditional cybersecurity and digital security by design. Their feedback was incorporated into the final version of cyber incidents which was further refined during the playtesting stage.

Players are not provided with any information on possible cyber-attacks before the start of the game and only begin discovering different attack scenarios as they progress through rounds. Once a cyber-attack card is drawn and dealt with, it is returned to the attack deck. This means that the same attacks can take place multiple times, which reflects the likelihood of cyber-attack repetitions in the real world.

#### Product Innovation

The game incorporates product innovation in the form of a four-stage product innovation tracker. Players must invest in product innovation to increase their product price, become more attractive to investors, and improve their chances of acquiring customers. To progress through the stages of the tracker, players must reach a sufficient level of product innovation investment (i.e., a sum of 50,000 in game money per stage) and meet the customer acquisition requirements (i.e., 5 new customers per stage). The concept of continuous product innovation was modelled into the game to investigate to what extent people prioritise product innovation under budget constraints and other investment requirements, such as cybersecurity and customer acquisition.

The product innovation board is inspired by the Levitt’s product life cycle (Levitt, 1965).^1^ The first stage represents market development, which is reflected in the lower product price and the lower probability of customer acquisition. The second stage represents the growth stage with increasing product price and customer acquisition probability, and the third and fourth stages represent maturity during which a business maintains a stable market share, but the product growth starts to slow down which is reflected in the lower customer acquisition probability in the third stage and the lower product price in the fourth stage. The lower product price can be also explained through the concept of economy of scale that businesses typically achieve at this stage (e.g., Bain, 1954).

#### Customer Acquisition

Customer acquisition remains a key component of a successful business growth strategy, as it initiates the customer lifecycle (Ang & Buttle, 2006; Drucker, 1973). *Threats and Trade-offs* incorporates a generalised idea of customer acquisition, according to which a company needs to invest in marketing for a chance to acquire customers. Players are provided with an opportunity to purchase customer acquisition attempts, which are represented as rolling dice in the game. The winning roll varies across different stages. In the first stage, teams acquire a customer only if they roll 1, which means that the probability of getting a customer is 16.7%. In later stages, the winning probability increases up to 50%. We have adapted the Diffusion of Innovation theory (Rogers, 2003) to determine the winning probability.^2^

Adding a mechanism for customer acquisition (dice roll) contributes to the complexity of the budget allocation dilemma set out in *Threats and Trade-offs*. Spending money on customer acquisition is not guaranteed to bring customers. Thus, we can model and observe the prioritisation of business development in relation to product innovation and cybersecurity investments.

#### Modelling the Firm’s Profit Function

The game assumes a player (or a team) to act as a profit maximising firm. The firm’s profit ${\pi }_{t}$ in each round $t$ is a sum of profits in the previous rounds ${\pi }_{t-1}$, external investment ${i}_{t}$, customer revenue, which is a product of number of customers ${n}_{t}$ and price the firm charges ${p}_{t}$, and minus total cost $t{c}_{t}$, i.e. any cost arising from a cyberattack or spending on business growth. Therefore, the simple round-to-round accumulated profit function is given by:

1 $${\pi }_{t}={\pi }_{t-1}+ {i}_{t}+{p}_{t}{n}_{t}- t{c}_{t}$$

Players can spend money on product innovation ${d}_{t}$ which cumulatively determines the business stage ${D}_{t}$, where$D\in \left[\mathrm{1,4}\right]$. External investment ${i}_{t}$ a function of ${D}_{t}$ and a random draw ${y}_{t}$ from a distribution of potential investors. Price ${p}_{t}$ is also a function of product development. Number of customers ${n}_{t}$ is a function of marketing efforts ${m}_{t}$ and the probability of success which is determined by${D}_{t}$. Customers accumulate from round-to-round, therefore:

2 $${n}_{t}={n}_{t-1}+ f({m}_{t},{D}_{t})$$

A player chooses marketing effort ${m}_{t}\in [\mathrm{0,5}]$ at a unit price ${q}_{t}$, which depends on the total number of customers.

Security measures can be purchased in any round at price $${w}_{t}$$ and accumulate over the game. The cost of a cyberattack $${c}_{t}$$ is equal to zero if the firm owns appropriate security measures $$\sum_{1}^{t}{s}_{t}$$ such that $$\sum_{1}^{t}{s}_{t}\ge {s}^{*}({a}_{t})$$, where $${s}^{*}$$ refers to the level of security that prevents attack $${a}_{t}$$ in round $$t.$$ Otherwise, $${c}_{t}$$ depends on the attack damage $${a}_{t}$$ and also frequently leads to loss of customer revenue from that round ($${p}_{t}{n}_{t})$$. Attack damage $${a}_{t}$$ is also a random draw from a distribution of possible cyber threats.

A successful cyber-attack can sometimes lead to reduction in customer and employee satisfaction, but these can be improved by investing money in customer satisfaction ${cs}_{t}$ and employee satisfaction ${es}_{t}$.

Players can take a loan ${l}_{t}$ in round $t$ with a simple interest rate $r$. The loan reaches maturity in round $j$ and it is then payable in full. The sum of loans taken out at some previous round $k,$ should it is payable now at $t$ is equal to $\sum_{k=1}^{t-1}[1+\left(t-k\right)r]{l}_{k}$

Therefore, the player or team is maximizing the complete objective function given by:

3 $${\pi }_{t}\left({d}_{t},{m}_{t},{s}_{t}\right)={\pi }_{t-1},\left({d}_{t-1},{m}_{t-1},{s}_{t-1},{s}_{t-1}\right)+{i}_{t}\left({D}_{t,}{y}_{t}\right)+{p}_{t}\left({D}_{t}\right){n}_{t}\left({n}_{t-1},{m}_{t}{D}_{t}\right)-{c}_{t}\left({\sum }_{1}^{t}{S}_{t},{a}_{t,}{m}_{t},{d}_{t}\right)-{m}_{t}{q}_{t}-{w}_{t}{s}_{t}-c{s}_{t}-e{s}_{t}$$

And is subject to the following constraint:

4 $${d}_{t}+ {m}_{t}{q}_{t}+ {w}_{t }{s}_{t }+ {cs}_{t}+ {es}_{t}\le {\pi }_{t-1} + {+ i}_{t}\left({D}_{t, }{y}_{t} \right) {+ l}_{t} -\sum_{k=1}^{t-1}[1+\left(t-k\right)r]{l}_{k}$$

For simplicity and readability, the firm’s profit ${\pi }_{t}$ in round $t$ is expressed only in terms of variables the player has control over: ${d}_{t},{m}_{t},{s}_{t}$, as the player chooses how much to invest in product development ${d}_{t}$, marketing efforts ${m}_{t}$ and cybersecurity ${s}_{t}$ and in terms of constraints there is a choice of a loan amount ${l}_{t}$. The game is a discrete instance of this model, and the winning conditions to be met at the final round $T$ are as follows:

5 $$\sum_{k=1}^{T}[1+\left(T-k\right)r]{l}_{k}=0$$

6 $${n}_{T}\ge 15$$

7 $${\pi }_{T}\ge \mathrm{200,000}$$

### Elements

**Fig. 3:**
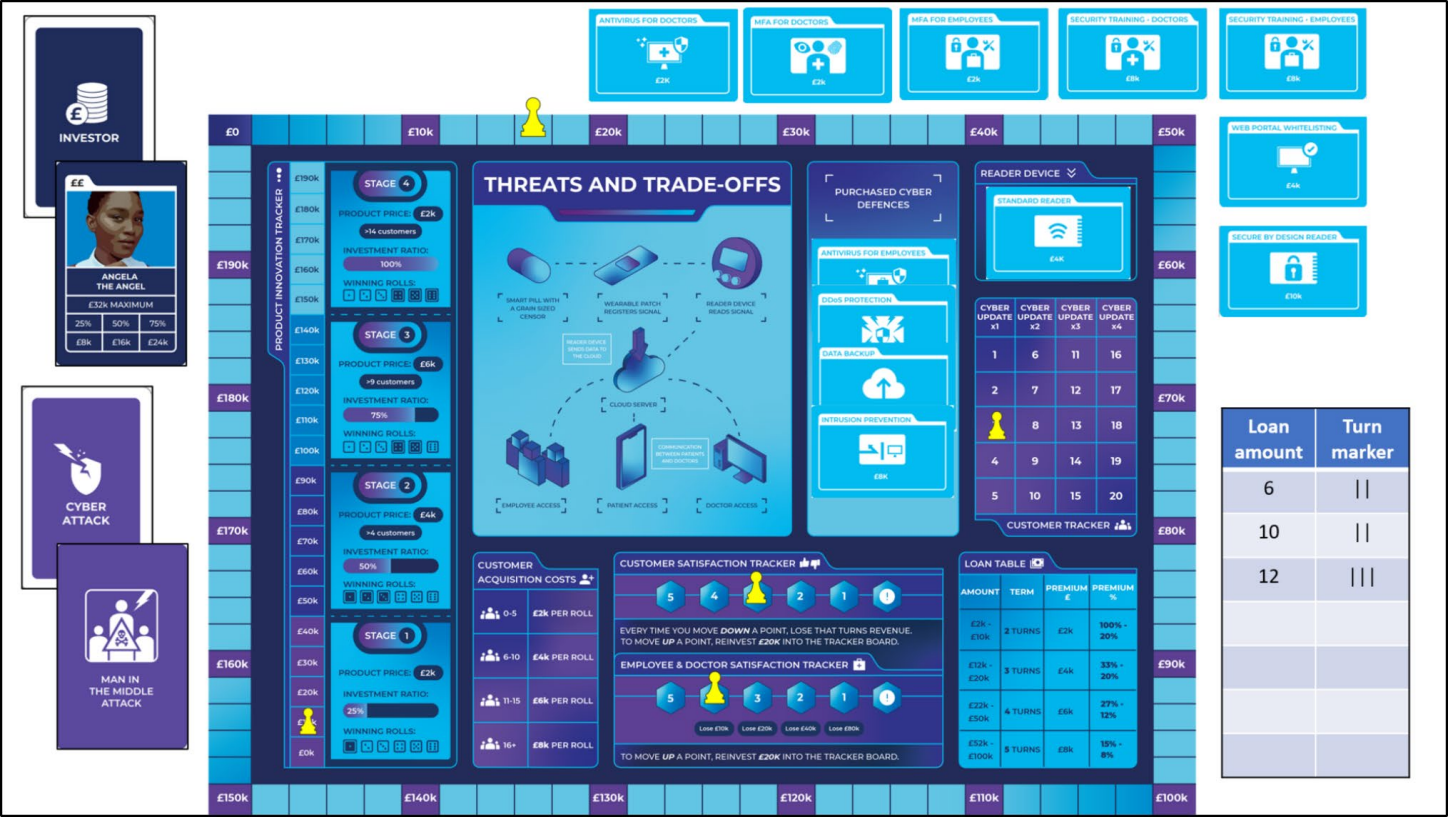
The game board with different elements

The game set-up includes a board, three card decks, five game counters (pawns or meeple), a die, and a loan tracking pad (Fig. 3). No real money is used in the game. A pound sign (£) has been used to communicate monetary value during game play. In the original version of the game, the Game Master (GM) has an information sheet with the cyber-attack scenarios and the associated mitigation measures and therefore does not take part in the game as a player. Furthermore, the authors offer a competitive version of the game on demand. The competitive version incorporates a cyber-attack deck with descriptions, mitigations, and outcomes of cyber incidents displayed on the cards, thus eliminating the need for a GM and making it possible for players to run the game independently. An online version of the game is also available at https://www.discribehub.org/boardgame-threats-tradeoffs. The description below focuses on the original version of the game, which is the base for the competitive and online versions.

At the start of the game, players are instructed to place counters to track different elements of their business on the game board, shuffle the “investor” and “cyber-attack” decks and place them face-down on the side of the board, and lay out the cyber defence cards face-up outside the board so that they are visible to all players. One of the players or the GM takes charge of the loan tracking pad.

#### Game Board

The game board has a “budget tracker” running along the edges. At the beginning of the game, a counter is placed on £0 and each square represents £2 k. As the team acquires funds, the counter is moved along the budget tracker. It is moved back when funds are spent. The game has two winning objectives: (a) to reach £200 k on the budget track with no outstanding loans on the loan pad and (b) to acquire 15 customers.

Teams also place a counter on the “product innovation tracker” at £0. The product innovation tracker indicates the current stage of the product lifecycle and shows how play conditions change in each stage. In addition, each stage of the product innovation tracker indicates the winning dice roll for customer acquisition, the product price that can be charged to the customers, and the investment ratio associated with that stage. The investment ratio represents the share of funds that players receive from an investor, with each individual investor card having its own maximum investment limit. Players can invest in product innovation (in multiples of £10 k) to enter a new product lifecycle stage and therefore increase the share of investor funds received, improve their chances of acquiring customers, and raise their product price.

A counter each is placed on the “customer satisfaction tracker” and the “employee and doctor satisfaction tracker”. At the beginning of the game, these counters are placed at 5, which indicates ‘extremely happy/satisfied’. However, the counters move down when a successful cyber-attack has negative consequences for customers or staff. The trackers go from 5 to 1, and if any of the counters fall below 1, players lose the game. To bring either of the trackers up by a point, players must invest £20 k into the respective tracker. Each decrease on the satisfaction trackers also represents monetary loss on the budget board. For example, if customer satisfaction goes down, the team does not receive customer revenue from that round.

The final counter is used for “customer tracker”. The customer tracker helps players to know when a cyber update is due. Cyber-defences once purchased remain in place until the next cyber update. At the point of updating, which takes place when the start-up’s customer base crosses a certain threshold, the team pays the original cost of the acquired defences if they want to keep them. Otherwise, the defences are returned to the market and made available for a future re-purchase at a higher price. With every cyber update, any unpurchased defences go up in price to reflect the increase in cybersecurity costs associated with business growth and development. Spending money on updating cyber defences incorporates the notion of technical and security debt (Rindell et al., 2019) which is considered inherent in operationalising software.

#### Cyber Defence Cards

**Fig. 4:**
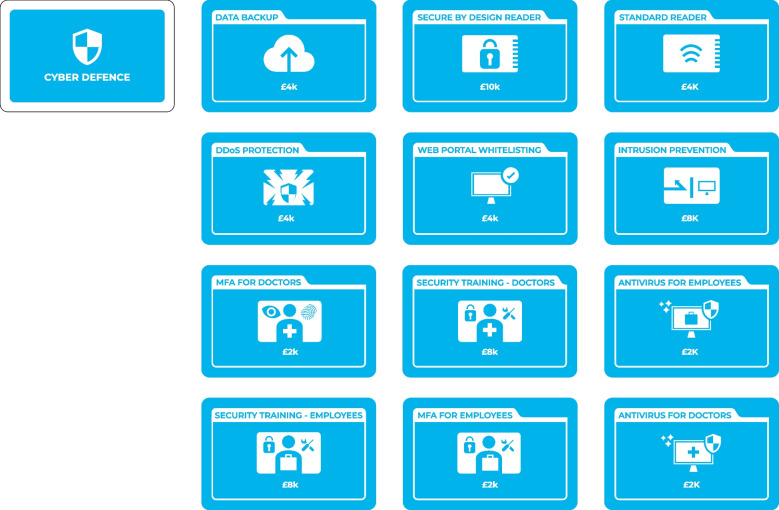
Cyber defence cards

Besides investing in product innovation, customer acquisition, and customer and employee satisfaction, players can use a turn to decide which cyber defences they would like to purchase. There are 12 cards which are placed face-up outside the board at the beginning of the game and brought into the board as and when they are purchased (Fig. 4). The market has cyber defences of different values. A team can get “antivirus” or “multifactor authentication” for as little as £2 k while “security training” for doctors and employees costs £8 k each. Some other measures (DDoS protection, Data Backup) cost £4 k. The costs of cyber defences also depend on the size of the organisation defined by the number of acquired customers. Once players have gained six customers, they need to repurchase previously acquired cyber defences at the base cost or return them to the deck. The prices of all unpurchased defences double. The same event occurs after players have acquired eleven and sixteen customers, with the respective cyber defence price coefficients of three and four. The game design makes it suboptimal to purchase all the defences, and there is an element of ‘smart choices’ to be made.

Additionally, cyber defences include two special cards that represent the base for the start-up's USP product – a reader device. The first option represents a standard reader that is commonly used in such devices. The second option represents a novel secure-by-design reader that provides a higher level of cyber protection than a standard device (Watson et al., 2015). However, it costs £10 k as compared to £4 k for the standard reader. Players cannot acquire customers until they purchase a reader device.

Finally, the cyber defences can be separated into three groups: those that affect the whole system (e.g., data backup, secure by design reader, and DDoS protection), those that protect employees (e.g., antivirus and security training for employees), and those that protect doctors (e.g., antivirus and security training for doctors). This added level of complexity reflects the diversity of initial access techniques that attackers use (e.g., MITRE ATT&CK®, n.d.) and demonstrates that in complex Internet-of-Things systems multiple vulnerabilities exist (both human- and technology-related).

#### Investor Cards

**Fig. 5:**
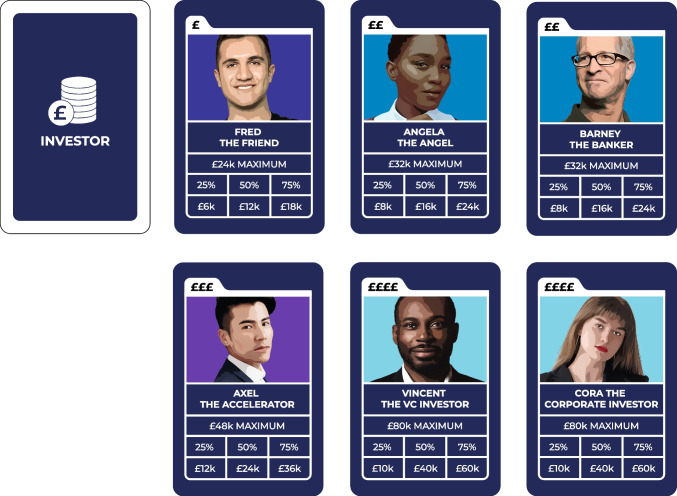
Investor cards

The investor deck is kept face-down and shuffled by the GM after every draw. The deck has 6 different investors with varying values (Fig. 5). For example, “Fred the Friend” can give £24 k at most while “Cora the Corporate Investor” has a maximum value of £80 k. Once a card is drawn, the funds received by the players are calculated as the ‘maximum investor value’ multiplied by the ‘investment ratio’. The investment ratio represents the share of available funds that the investor is willing to give. The ratio is indicated on the product innovation tracker and increases with each new product lifecycle stage. After a card is drawn, it is returned to the deck, so the probability of drawing the cards stays the same.

After drawing an investor card, players can decide whether they want to take a loan or not. A loan comprises of a principal and interest, and the principal cannot exceed the current balance on the budget tracker. Each loan has a term reflected in the number of turns before the loan becomes due for repayment. Interests and terms depend on the loan sum, and the respective information is provided in the “loan table” on the main board. Loans must be repaid in full before the term expires. For instance, if a loan was taken out in the turn 1 of the game, and it has a term of 3 turns, it will be due for repayment at the end of the turn 3.

The types of investors were compiled based on journalistic sources (e.g., Cremades, 2019) and practical guides on startup series funding (e.g., Boxall, 2023; Rutan, 2023). Additional sources were used to estimate relative investment sizes of each investor type (e.g., Bone et al., 2017; British Business Bank, n.d.; British Business Bank, 2018; FundersClub, n.d.), which in turn informed investor categories in the game. During a series of playtests, the investment sizes of each investor card were adjusted to fit the game mechanics while still maintaining the conceptual representation of the associated investment type relative to the business context. A project-external innovation expert was consulted to confirm the viability of the identified investment categories. It is also important to notice that while the game aims to educate players on general startup investment opportunities and options, together with other aspects of business management such as product innovation and customer acquisition, this was not the key focus of the game, and the investor types were selected to support the game mechanics of receiving game money to facilitate player decision-making and budget allocation.

#### Attack Deck

**Fig. 6:**
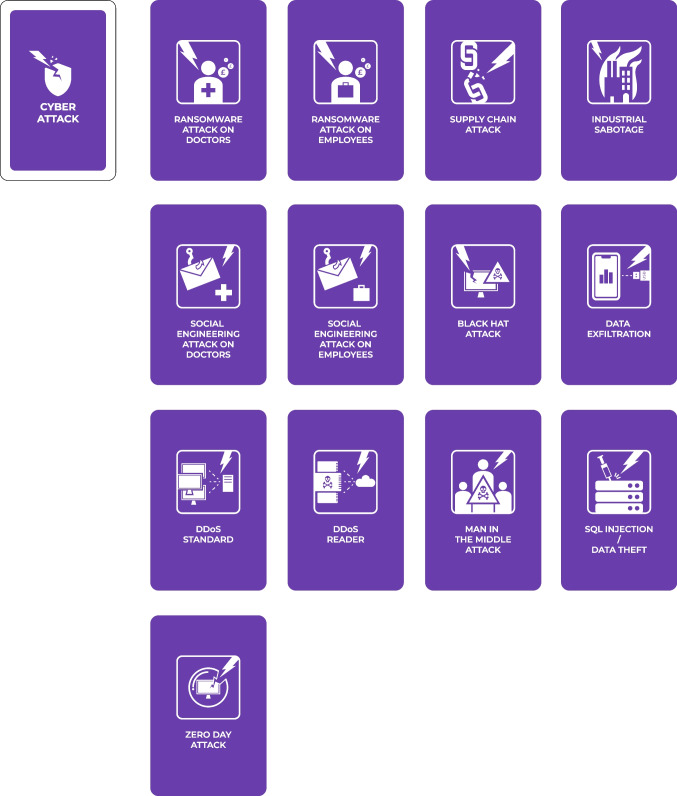
Cyber-attack cards

To end a turn, players draw from the face-down cyber-attack deck and show the card to the GM who consults the cyber-attack scenario sheet and checks if the right defences are in place. If the attack is mitigated, the GM provides a brief description of the attempted cyber-attack but does not reveal any essential information, such as required defences or potential consequences. If the correct defences are not in place, besides describing the cyber-attack, the GM informs players about the consequences and enforces the associated penalties. There are 13 cyber-attack cards in the deck (Fig. 6), and once a card is drawn, it is shuffled back into the deck. This means that players can face the same cyber-attack again. The potential repeatability of cyber-attacks distinguishes our game from other cybersecurity games like *Decisions and Disruptions*.

In addition, the cyber-attacks can be separated into three groups: those that affect the whole system (e.g., supply chain, industrial sabotage, and DDoS), those targeted at employees (e.g., social engineering and ransomware attacks on employees), and those targeted at doctors (e.g., social engineering and ransomware attacks on doctors). This added level of complexity reflects the diversity of initial access techniques that attackers use (e.g., MITRE ATT&CK®, n.d.) and demonstrates that in complex Internet-of-Things systems multiple vulnerabilities exist (both human- and technology-related).

The consequences of cyber-attacks include loss of customers, decrease in customer satisfaction, decrease in doctor and employee satisfaction, and the base fine of £10 k. Some penalties are only incurred if the company has at least one customer. Additionally, the fines depend on the size of the organisation defined by the number of acquired customers. Once players have gained six customers, the cost of penalties is doubled. The same principle holds after players have acquired eleven and sixteen customers, with the respective penalty coefficients of three and four.

If the outcomes of a cyber-attack put the budget below 0, players are given an opportunity to draw from the investor deck to make up the balance. If the received investor funds are not sufficient to bring the budget balance to 0 at least, players can make another attempt by taking out a loan. In this case, the maximum loan value is calculated as the sum of the budget tracker balance before the cyber-attack and the received investor funds. If the budget tracker balance remains below 0 after drawing an investor card and taking out a loan, players lose the game.

The survival of the cyber-attack marks the end of the turn, and the remaining budget balance carries over to the next turn. At this point, players repeat all the previous steps until they either win or lose the game.

The exact turn order and the rules of the game are laid out in the GM guide, available for download at https://www.discribehub.org/boardgame-threats-tradeoffs.

## Evaluation and Testing

T&T is a tabletop board game where players collaboratively make decisions to expand a new smart healthcare start-up while confronting the challenges of budget constraints, taking chances on marketing for customer acquisition, investing in product innovation, and avoiding cyber-attacks. The game simulates the challenges of running a start-up, managing investments and debts, and making choices under incomplete information. The cyber scenarios used in the game create learning triggers to acquaint players with different types of cyber-attacks, their consequences, and countermeasures.

The game was first played by 32 students enrolled in a computer science course at a British university during a scheduled lecture session on risk management. The game was utilised in addition to the established programme curriculum, with the aim of introducing students to general organisational risks and consequences of cyber-attacks. While the game does not directly employ any specific approaches to risk management, in can be used in conjunction with those to provide an immersive learning experience. The game provides a generalised overview of the organisational structure and data flow, which can be used to practice high-level risk management processes. Furthermore, to demonstrate the general risks and consequences of cyber-attacks to an organisation, the game incorporates the following elements and mechanics: a decrease in employee and doctor satisfaction, a decrease in customer satisfaction, loss of customers, fines, and other financial penalties—all of which can ultimately lead to losing the game (e.g., going bankrupt).

During the lecture, seven teams played the game, and seven Game Masters were involved in delivering the game session. The Game Masters were members of teaching and research staff at the university and had been briefed about the game and its rules prior to the game session with students.

Participants were informed that the gameplay would be audio-recorded, and the audio transcripts would be anonymised prior to analysis. An inductive approach was used to analyse the transcripts due to the unstructured nature of audio data consisting of recorded player interactions and discussions during the course of the game. All teams were offered a debrief at the end of the game which produced some post-game reflections that were also coded while conducting content analysis of the transcripts. Five themes emerged from content analysis – learning about cybersecurity, learning about business development, motivations for investing in cybersecurity, player enjoyment, and semblance of reality—which we place in two categories of evaluation: outcomes and processes. Players were also asked to fill out a short questionnaire at the end of the game (see Appendix) which allows us to provide quantitative indicators for some of the themes which emerged.

### Outcomes

We observed that playing the game created ‘learning triggers’, not just for cybersecurity, but also for business and management concepts. These were often expressed in conversations with the Game Master or conversations between the players themselves. The audio transcripts also captured different decision-making logics and reasoning behind cybersecurity investment, and some of the players reflected on them more substantively after the game.

#### Refresher on Cybersecurity

One of the main outcomes of playing the game is learning about cybersecurity threats. Playing T&T created an opportunity for players to refresh their memory on different cyber-attacks and possible mitigation strategies. An excerpt from one of the teams is presented below:

*Game Master: Okay, so we’ve got three customers. We’ve done all of our investments and purchasing; no product investments. So, we’ve got another cyber-attack underway. And it’s a zero-day attack.*

*Player: Oh no. Bad, it’s very bad. Spoilers – it’s quite bad. It’s also* [unclear]*, as in on the back. Is it on the back?*

*Game Master: There’s no mitigation for it.*

*Player: Yes, that’s what I thought.*

From the participant survey, we note that self-reported cybersecurity knowledge scores, on a scale from 0 to 10, ranged from 2 to 9, with a mean of 5.438 (Table 1). The players themselves highlighted how useful cybersecurity awareness was for playing the game, but at the same time noted that a lack of knowledge was not a barrier as the game allowed players with differing levels of knowledge to learn during the game. This is captured in an exchange between two players below:

*Player 1: This game, only people with cybersecurity knowledge can play it, actually.*

*Player 2: Not really.*

*Player 1: A lot of people don't understand any of these things, you know?*

*Player 2: Yeah, but you find out, because during the attack, this protects this.*

#### Learning About Business Development

Another outcome of playing the game is gaining knowledge of business development and entrepreneurship. From Table 1, we note that the average self-assessment of entrepreneurial skills was lower than cybersecurity skills. While the players with their background in computer science found it easy to grasp the cybersecurity aspects of the game (an attack, its implications, and countermeasures), it was interesting to observe that they required a more extensive explanation of the business development elements of the game. For instance, it took players some time to understand and contextualise the link between their current budget and the maximum loan limit.

The game allowed players to experience different aspects of running a business, including the monitoring and maintenance of customer satisfaction. Some cyber-attacks reduced customer satisfaction, and to bring it back up again, players had to make a substantive investment (£20 k). There was an incentive to keep customer satisfaction high because the game ends if it falls below 1. One player remarked that the customer satisfaction tracker made them realise that “reputation is everything” in a business.

#### Investing in Cybersecurity

Teams displayed a variety of investment strategies – some teams drew on their knowledge of digital security measures and benefitted from early investments in cybersecurity, while others adopted a more reactive approach. An immediate trade-off that teams faced was between investing in cybersecurity (which would prevent financial losses from cyberattacks) or investing in customer acquisition (which would bring revenue). Both these choices were associated with uncertainty – cyber-attack cards are randomly drawn at the end of the round and customers are acquired based on a dice roll. It is important to notice that players would not be able to win the game without balanced investment in all three aspects: cybersecurity, product innovation, and customer acquisition.

We noticed that many teams were apprehensive about investing in customer acquisition early due to the low probability of a winning dice roll and the cost associated with each attempt, even though the reader device was often the first thing they purchased. One of the teams had a strategy of rolling the die once every round, whereas some other teams waited for a few rounds before rolling the die five times in one go. One of the players noted that their strategy was to prioritise cybersecurity over customer acquisition because *“I think it’s more important to cover your bases before you have a large customer base than the other way round.”* A post-game reflection echoed this point:

*“So we did try and getting* [sic]* customers on board but then because of cyber-attacks, we lost a few customers. But then we thought that we should invest more on the security and the organisation so that we didn’t hamper our customers.”*

One of the player’s reasoned that cybersecurity investment was motivated by the dependence of the business on the digital world:

*“Just the new world, everything is on the cloud, everything..., mobile apps, so there is the cloud for that or the servers, so it’s all digital. Any start-up you see, it’s like everything’s gone digital so you need protection security systems or else you lose customers, you lose revenue, and you lose business.”*

At the same time, some players were less risk-averse and displayed the recency bias in their decisions to invest in cybersecurity, i.e., decisions to purchase security measures were informed by previous cyber-attacks.

*Player 1: Okay, so that last attack we needed… what would have helped us, the multifactor authentication?*

*Player 2: Yeah. I think that’s a good option.*

Another excerpt of interactions highlights the optimism bias (with author emphasis added):

*Game Master: You still have two* [security]* measures that you haven’t bought, just as a reminder.*

*Player 1: Ah, we’ve been lucky for now.*

*Player 2: I think we don't have to pay more right now.*

*Player 3: For Employees.*

*Player 2: Employees. How much does it cost, 2k?*

*Player 3: No, 8k.*

*Player 1: No, bro. Leave those for now.*

*Player 3: I think we should buy one of them.*

*Player 1: No, man.*

*Player 3: Are you sure?*

*Player 1: I'm positive. When have we needed any of those yet? We haven’t needed them yet. We’ve been lucky with the draws.*

Six of the seven teams purchased the standard reader first and eventually purchased the secure-by-design reader which provided additional security. The round for purchasing the more secure device ranged from 3 to 12. Teams with higher initial budgets found it easier to invest in cybersecurity and choose the secure-by-design reader (2.5 times more expensive). Even so, a conscious decision had to be made about choosing the more secure reader, as can be noted from a player’s reasoning below:

*“It* [cyber-attack] is risky and you might lose customers, and this is all about cyber security so we should probably go for the secure one. And if we have a secure reader, probably we don’t need a standard reader, yeah? So, we can save us money or else we’ll have to get it later probably. So, I think *since we have good money we should go for secure design.”*

### Process Evaluation

Two other themes were noted from the transcripts – player enjoyment and semblance of reality – which relate closely to learning and research. Player enjoyment makes games engaging tools to facilitate learning and research participation. The ability of a game to simulate real life makes it possible to observe human behaviour in a controlled environment. While any simulation is an approximation of reality, it helps to uncover behavioural and decision-making patterns that occur in the “real world”.

#### Player Enjoyment

It was encouraging to observe that most players found the game enjoyable, which is demonstrated by statements like “I definitely got right into this” in the post-game reflection. One of the players commented that the game may be “a good team-building thing”, alluding to the social aspect of games and game-based learning (Denning et al., 2013). During the debrief, players were asked if they had any other comments about the game to which most of them responded that they found the game ‘interesting’, ‘fun’, and ‘a good experience’. The excerpt below suggests that participants found the game both educational and enjoyable.

*Player 1: It was enjoyable, yes.*

*Player 2: It was really interesting.*

*Player 3: Even though it was a game, we learnt a lot about the* [business development] *stages and then the kinds of attack, what we needed to mitigate those attacks.*

In the survey, the average response to the question “How much did you like the game?”, where 0 means ‘hated it’ and 10 means ‘loved it’, was 8.8. Similarly, responses to the question “Was the game boring (0) or terrific (10)”, ranged from 5 to 10, with an average value of 8. Players displayed a larger variation to other questions about the game design like its complexity, playing time, and number of choices or options available during game play (Table 2). However, we also noted that appreciation of the game’s complexity is positively correlated to perceiving the game as more ‘skill-based’ than ‘luck-based’ (Table 3).

**Table 2:** Post-game evaluation by players

Variable	Obs	Mean	Std. Dev	Min	Max
Complexity (0 = very simple, 10 = very complex)	32	6.188	2.375	3	10
Game Instructions/Rules (0 = very simple, 10 = very complex)	32	5.594	2.227	1	10
Luck vs. Skill (0 = pure luck, 10 = all skill)	32	5.906	1.422	2	9
Uniqueness/Game Mechanics (0 = not much different, 10 = very different)	32	6.563	2.124	2	9
Playing time (0 = too short, 10 = too long)	32	6	1.901	1	10
Game Idea (Concept) or Theme (0 = boring or weak, 10 = terrific)	32	8	1.626	5	10
Interest (0 = hated it, 10 = loved it)	32	8.813	1.33	6	10
Game Options (0 = not enough, 10 = too many)	31	6.419	2.277	1	10

**Table 3:** Game attributes—correlation table

Variables	(1)	(2)	(3)	(4)	(5)	(6)	(7)	(8)
(1) Complexity	1.000							
(2) Game Instructions/Rules	0.674***	1.000						
	(0.000)							
(3) Luck vs. Skill	0.320*	0.405**	1.000					
	(0.074)	(0.021)						
(4) Uniqueness/Game Mechanics	0.055	− 0.032	0.103	1.000				
	(0.764)	(0.862)	(0.573)					
(5) Playing time	0.379**	0.373**	0.406**	0.423**	1.000			
	(0.033)	(0.035)	(0.021)	(0.016)				
(6) Game Idea/Theme	0.067	− 0.071	0.014	0.168	0.417**	1.000		
	(0.716)	(0.698)	(0.940)	(0.358)	(0.017)			
(7) Interest	0.124	− 0.005	− 0.061	0.187	0.191	0.611***	1.000	
	(0.500)	(0.979)	(0.741)	(0.306)	(0.294)	(0.000)		
(8) Game Options	0.439**	0.284	− 0.018	0.146	0.602***	0.338*	0.144	1.000
	(0.013)	(0.121)	(0.925)	(0.434)	(0.000)	(0.063)	(0.440)	

*** *p* < 0.01, ** *p* < 0.05, * *p* < 0.1

#### Semblance of Reality

Games occupy a liminal space with respect to depicting reality. While rules of play and consequences can mimic real life and games are often situated in real-life contexts (managing a business, building a society, completing a mission), they are simulations with certain fixed assumptions, much like theoretical or statistical modelling. With T&T, some teams found that the game was immersive and true to life.

*Game Master: … Did you find it [the game] realistic to some extent?*

*Player 1: Yeah, because… I don’t know, you feel like with a start-up you are always borrowing money to stay afloat, and that’s what we were going quite a lot. And that replicated… but then you can turn it into profitability quite quickly, so from our customers, once we get to this stage, it’s quite easy to gain customers and we were getting a lot of money from it when we got to stage 3. So I do think it’s somewhat realistic.*

*Player 2: And then the fact that we defend ourselves from all the potential threats available.*

*Game Master: Yeah, a lot of them out there.*

*Player 2: Yeah, made sure that we didn’t lose money from like compliance or like actual data loss, or whatever it is.*

On the other hand, during the post-game debrief, one of the players pointed out that their strategy was affected by the game mechanics and might have been different in real life. Author emphasis has been added to highlight the points where the player considers the game to diverge from reality.

*Player: I think our strategy was solely based on the fact that we were thinking of it as a game, as opposed to a real-life scenario. If it was real life, I personally wouldn’t just start buying all these cyber additions, because you’ve got to understand, I'm assuming if we’ve got no customers, there’s no real incentive for attackers to get us, what data can they get really? So, they would be going for our competitors. So, I would probably focus on acquiring customers, and then once then there is an incentive for attackers, then I would start incorporating cybersecurity.*

This is also related to the discussion about when to invest in cybersecurity (c.f. 4.1.3). There are benefits to investing in cybersecurity early in the game, and arguably in real-life as well. Start-ups are vulnerable to cyberattacks and face financial, reputational, and regulatory implications (Selamat et al., 2022), and perhaps the above reflection can be ascribed to optimism bias or lack of awareness. For example, the Cyber security breaches survey 2023 in the UK identified that 11% of businesses had experienced cybercrime in a 12-month period (DSIT, 2023). We therefore argue that a key contribution of T&T as a game lies in challenging commonly held beliefs about who is vulnerable to cyber-attacks and discussing, modelling, and understanding beliefs about cyber-threats and risks, optimality of associated cybersecurity investment.

## Discussion and Conclusion

The expansion of the digital world makes cybersecurity a critical issue. For businesses, this digital expansion requires investing in cybersecurity which leads to short- and medium-term trade-offs as limited budgets have to be diverted from other parts of the business, such as product development or marketing. We have created a boardgame-based simulated environment that highlights this dilemma and focusses on the experiences of start-ups or small firms (SMEs). It is particularly important to focus on SMEs since they are the main constituents of the private sector (99% of private sector businesses in the EU and 99.9% in the UK are SMEs) and they also keenly experience the costs of cybersecurity, which Heidt, et al. (2019) characterise as the “security divide”.

Our game serves two purposes. Firstly, it is an educational tool, and unlike other games which exclusively focus on building cybersecurity awareness or cybersecurity skills, *Threats and Trade-offs* interweaves cybersecurity with business development. From our playtesting, we found that the game made it possible for postgraduate computer science students to learn something new about business development, debt management, and reputation management. The players themselves remarked that the game was also helpful in learning about cyber-attack scenarios and associated defences, and while prior knowledge is an advantage, it is not a barrier to game play itself. The students found the game engaging and playing as a team added a socialising or team-building element into the classroom learning environment. The interdisciplinary nature of the game helps to build knowledge about cybersecurity and business development and addresses learning needs in multiple subject areas.

Secondly, the game serves as a research tool for understanding how groups make decisions about allocating limited budgets to cybersecurity investment. We noticed both reactive and proactive approaches to cybersecurity. The reactive approach was demonstrated by decision-making based on previous cyber-attacks (recency bias) and optimism bias. The proactive approach was demonstrated by some teams who diverted funding from customer acquisition to purchasing cyber-defences, although this was mainly driven by fear of cyber-attacks and the severity of its repercussions rather than any inherent positive value of security. The initial investor cards, which are also drawn at random, affected financial ability to choose the secure-by-design reader as opposed to the standard reader.

Building a boardgame or a simulation explicates the assumptions we make about an environment, which then allows a discussion about how realistic or unrealistic those assumptions are. Some of the players who played *Threats and Tradeoffs* argued that it is unrealistic to expect that a start-up would face cyberattacks, although this is not the case and biotech startups in particular have been targeted in the recent past (Smith, 2021). Arguing that a small start-up is not going to be a target for cyber-attacks echoes the optimism bias and conundrum of people believing that they would be highly vulnerable in case of a cyber-attack while also believing that they are unlikely to be targeted (de Smidt & Botzen, 2018). The game can thus be a vehicle for challenging this assumption and contribute to the wider goal of rationally and realistically assessing the value of cybersecurity and the investments required.

We intend to conduct more game sessions and record decision-making with a baseline version of the game (described here) and some altered mechanics or treatments. For example, how are cybersecurity investment decisions affected in a market with limited number of customers, or in the case of network effects (a cyberattack on one team affects everyone else in the room), or time constraints? The networked nature of the digital world results in a number of externalities which may lead to underinvestment in cybersecurity and market failures. Alternatively, the regulatory environment could be altered in order to vary the incentives and requirements for information security and data protection. A better understanding of how collective decisions on cybersecurity are made can help in unpacking the enablers and barriers faced by start-ups and SMEs with respect to cybersecure hardware adoption and cybersecurity investment. This knowledge could then inform the targeting and marketing of cybersecurity solutions as well as government policy and support.

The resources associated with the game (printable materials) can be made available upon request and are covered by CC BY-NC licence, i.e., the work can be tweaked and built upon for non-commercial use like teaching and research.

## Data Availability

Playtest data and study materials are available on request from the corresponding author. Game materials are available for download on https://www.discribehub.org/boardgame-threats-tradeoffs.

## Appendix

Table 4.

**Table 4:** Cyber-attack scenarios

Cyber-attack	Description	Cyber-defence	Cyber-harms
Supply chain attack	There has been a cyber-attack on your pill supplier. Unfortunately, there was no way for you to prevent this attack, but the attack has a carry-over consequence for you	No mitigation	Customer satisfaction tracker goes down a point
Black hat attack	A black hat breaks into your reader device, exploiting a memory vulnerability and causing it to malfunction	Secure-by-design reader device	Customer satisfaction tracker goes down a point
Industrial sabotage	A competitor pays a cyber-crime facilitator to exploit memory-based vulnerabilities in your reader devices and causes them to malfunction	Secure-by-design reader device	Customer satisfaction tracker goes down a point
Data exfiltration	An attacker finds a memory-based vulnerability in your reader devices, exfiltrates data from a large number of devices and sells it on a dark web. A cybersecurity researcher finds this data and informs you and the government	Secure-by-design reader device	Customer satisfaction tracker goes down a point. Fine of 10 k
DDoS through reader device	An attacker finds a memory-based vulnerability in your reader devices and exploits it to execute a DDoS attack from the devices’ IP addresses	DDoS protection OR Cheri-based reader device	Employee and customer satisfaction trackers go down a point
DDoS attack on the cloud	An attacker executes a DDoS attack on your cloud	DDoS protection	Employee and customer satisfaction trackers go down a point
Phishing/ransomware attack on employees	An attacker sends out phishing emails with a malicious link to your employees. A senior employee clicks on the link, activating the ransomware that blocks access to the key components of your network	Employee training OR Antivirus for employees OR Data back up	Employee satisfaction tracker goes down Ransom payment—20 k *If paid, the game master decides whether the access is restored* *If the ransom is not paid, or paid but the access is not restored, move the customer satisfaction tracker down a point*
Phishing/ransomware attack on doctors	An attacker sends out phishing emails with a malicious link to your doctors. A doctor clicks on the link, activating the ransomware that blocks access to the key components of your network	Doctor training OR Antivirus for doctors OR Data back up	Customer satisfaction tracker goes down Ransom payment—20 k *If paid, the game master decides whether the access is restored* *If the ransom is not paid, or paid but the access is not restored, lose double the revenue*
MITM/IP theft	One of your employees has been working from a coffee shop using unsecure public wifi. An attacker has made a connection to your employee’s device and managed to steal sensitive information that he used to obtain your company’s IP, which he later sold to a competitor. Some of your customers switched to your competitors’ services	Employee training	Lose a customer
SQL injection/Data theft	An attacker uses a vulnerability in your web portal to make an SQL injection and steal patients’ data. A cybersecurity researcher finds the data on the dark web and reports the data breach to the authorities	Web Portal Whitelisting OR Intrusion Prevention System	Customer satisfaction goes down a point Fine – 10 k
Social engineering/data theft attack on doctors	An attacker executes a pretexting social engineering attack on one of your doctors, obtains their credentials, and steals sensitive patient data	MFA for doctors OR Intrusion Prevention System OR Doctor training	Customer satisfaction goes down a point Fine – 10 k
Social engineering/data theft attack on employees	An attacker executes a pretexting social engineering attack on one of your doctors, obtains their credentials, and steals sensitive patient data	MFA for employees OR Intrusion Prevention System OR Employee training	Employee satisfaction goes down a point Fine – 10 k

## Footnotes

1 However, for the purposes of the game, we excluded the decline stage from our product innovation board. Instead, we split the growth stage into two consecutive stages that reflect the integration of a novel product into the market.

2 In the first stage, only a small segment of the population who are ‘early adopters’ and ‘innovators’ purchase the new product or service being offered by the start-up. In the second stage, the share of population interested in the novel product expands to the ‘early majority’, who tend to adopt innovation before the average person but also need to see some evidence, examples, and success stories before committing themselves. In stage 3, we assume that the pool of potential customers is shrinking as ‘early adopters’ lose interest and start looking for the next new thing. This makes the winning probability fall from 50 to 33% (i.e., ‘late majority’). By stage 4, we assume that the start-up is attracting both ‘late majority’ and ‘laggards’, and thus the winning probability rises back to 50%.
